# Visual word recognition: Evidence for a serial bottleneck in lexical access

**DOI:** 10.3758/s13414-019-01916-z

**Published:** 2019-12-12

**Authors:** Alex L. White, John Palmer, Geoffrey M. Boynton

**Affiliations:** 1 Institute for Learning and Brain Sciences, University of Washington, Seattle, WA USA; 2 Department of Speech and Hearing Sciences, Institute for Learning and Brain Sciences, University of Washington, 1715 Columbia Rd NE, Box 357988, Seattle, WA 98195 USA; 3 Department of Psychology, University of Washington, Seattle, WA USA

**Keywords:** Visual word recognition, Attention: divided attention and inattention, Attention: theoretical and computational models

## Abstract

Reading is a demanding task, constrained by inherent processing capacity limits. Do those capacity limits allow for multiple words to be recognized in parallel? In a recent study, we measured semantic categorization accuracy for nouns presented in pairs. The words were replaced by post-masks after an interval that was set to each subject’s threshold, such that with focused attention they could categorize one word with ~80% accuracy. When subjects tried to divide attention between both words, their accuracy was so impaired that it supported a serial processing model: on each trial, subjects could categorize one word but had to guess about the other. In the experiments reported here, we investigated how our previous result generalizes across two tasks that require lexical access but vary in the depth of semantic processing (semantic categorization and lexical decision), and across different masking stimuli, word lengths, lexical frequencies and visual field positions. In all cases, the serial processing model was supported by two effects: (1) a sufficiently large accuracy deficit with divided compared to focused attention; and (2) a trial-by-trial stimulus processing tradeoff, meaning that the response to one word was more likely to be correct if the response to the other was incorrect. However, when the task was to detect colored letters, neither of those effects occurred, even though the post-masks limited accuracy in the same way. Altogether, the results are consistent with the hypothesis that visual processing of words is parallel but lexical access is serial.

**Electronic supplementary material:**

The online version of this article (10.3758/s13414-019-01916-z) contains supplementary material, which is available to authorized users.

## Introduction

When listening to a story, the sensory signal is defined by change across time, and the words are presented sequentially. But when reading a story, the sensory signal is defined by change across space, and many words are available simultaneously. The visual system is capable of parallel processing across space, starting with the simultaneous retinal transduction of the entire incoming image. Therefore, it is theoretically possible that multiple written words can be processed in parallel.

The degree of parallel processing in natural reading is the subject of a long-running debate. The debate has been mostly fueled by measures of oculomotor behavior. For instance, readers fixate the majority of words directly, but they begin processing the next word (*n+*1) while still fixating on the current word (*n)* (Rayner, 2009). That can be shown by surreptitiously changing word *n+1* during the saccade to it, which results in a slowdown of processing in the next fixation. But does that mean the two words (*n* and *n+1*) were processed in parallel? Some researchers argue affirmatively, based on a range of experimental data fit with computational models (Engbert, Nuthmann, Richter, & Kliegl, 2005; Snell, van Leipsig, Grainger, & Meeter, 2018b). Others argue, to the contrary, that word recognition is necessarily serial: attention shifts to begin processing word *n+1* only after word *n* is completed (Reichle, Liversedge, Pollatsek, & Rayner, 2009; Reichle, Pollatsek, & Rayner, 2006).

The debate has recently extended beyond oculomotor measures during reading (Snell & Grainger, 2019b). For instance, several studies have shown that with relatively short displays (≤ 200 ms) word recognition performance is influenced by surrounding words and sentence context (Snell, Declerck, & Grainger, 2018a; Snell & Grainger, 2017; Snell, Meeter, & Grainger, 2017). This could be taken as evidence that multiple words are processed in parallel, although questions remain about the precise temporal dynamics of multiple word recognition.

In two recent studies, we took a related approach to ask a fundamental question: Can people recognize two words at *exactly* the same time? We used backwards masking to control the amount of time available to process each word. Specifically, we presented subjects with pairs of nouns, one to the left and one to the right of fixation. The nouns were flashed briefly and immediately preceded and followed by masks of random consonants. There were two main conditions: (1) In the *single-task* condition, the subject was pre-cued in advance to the location of the one word they had to recognize, so they could focus attention on it and ignore the other (while fixating centrally). (2) In the *dual-task* condition, the subject was pre-cued to both locations, so they had to divide attention and try to recognize both words simultaneously. At the end of the trial they were prompted to judge both words independently. In both conditions, the subject had to report whether each attended word belonged to a specific semantic category (e.g., “animals”).

Importantly, we set the duration of the inter-stimulus intervals (ISIs) between the words and the masks to each subject’s threshold, such that in the single-task condition they could categorize one word with ~80% correct accuracy. The question was, in that same amount of time, could they recognize both words? The answer was no: with the same stimulus timing, in the dual-task condition accuracy was sufficiently degraded that it ruled out two standard parallel models and supported an “all-or-none” serial processing model. This serial model assumes that only one word can be fully recognized at a time, and due to the limited time available, only one word can be recognized on each trial. If the subject is asked about the other word, they have to guess. Hence the name “all-or-none”: each word is either processed completely, or no task-relevant information is extracted at all.

We also found a trial-by-trial stimulus processing tradeoff in the dual-task condition: subjects were more likely to respond to one word correctly if they responded *incorrectly* to the other word. This tradeoff pattern also suggests that the subjects can’t recognize both words on each trial, and therefore provides further support for the all-or-none serial model.

However, when subjects viewed exactly the same stimulus sequences but had to judge the color of the letters, rather than the meaning of the words, dual-task accuracy was equivalent to single-task accuracy. Each dual-task response was more likely to be correct if the other was correct, unlike the stimulus processing tradeoff pattern we observed in semantic judgments. Overall, color detection performance was consistent with unlimited-capacity parallel processing, while semantic categorization performance suggested that a serial bottleneck lies somewhere in the word recognition system (White, Palmer, & Boynton, 2018).

In a subsequent study, we investigated the source of that bottleneck in the brain’s reading circuitry. We recorded brain activity with fMRI while participants performed a semantic categorization task with masked words to the left and right of fixation, similar to the experiment described above (White et al., 2018). We observed evidence of parallel processing of the two words throughout visual cortex. But in an anterior sub-region of the left hemisphere “visual word form area,” activity was consistent with serial processing of single words (White, Palmer, Boynton, & Yeatman, 2019).

In the experiments reported here, we sought to answer five of the questions left unanswered by our previous studies. First, is the serial bottleneck specific to high-level *semantic* judgments, or does it apply to any task that requires lexical access? Lexical access is the stage at which a written word activates an entry stored in long-term memory. Lexical access is often studied using the lexical decision task: the subject is presented with letter strings and reports whether they are real words or not. No further semantic processing is required. In Experiment 1, we assessed parallel vs. serial processing with a semantic categorization task (distinguishing living things from non-living things), and Experiment 2 we used we use a simpler lexical decision task (distinguishing real English words from pseudowords).

Second, is the serial bottleneck specific to words presented in opposite hemifields? With one word in the left hemifield and the other in the right, we previously observed a marked asymmetry: semantic categorization accuracy was much higher for words to the right than left of fixation (White et al., 2018), consistent with a many decades of prior studies (e.g., Mishkin & Forgays, 1952). It is possible that the inherent asymmetry induced a strategy of only attending to the right word in the dual-task condition. Therefore, in the three experiments here, we presented the words directly above and below fixation. Accuracy for those two locations is more balanced, and the letters are all closer to fixation and easier to resolve.

Third, is the serial bottleneck apparent only for some types of post-masks? Our prior results may have depended on masks composed of letters that caused interference at the level of orthographic processing. In Experiment 1, we directly compared two different masks: letters, and noise patches made by phase-scrambling images of letters. The scrambled masks were matched to the letters in spatial frequency and orientation content, size, and luminance contrast, but contained no objects. In Experiments 2 and 3 we used upside down non-letter characters as masks. These masks were composed of letter-like features arranged into objects that nonetheless aren’t recognizable letters.

Fourth, can two words pass through the bottleneck together if they are very short and common in the language? Short and common words may require fewer processing resources and therefore be processed in parallel. To test that possibility, in all three experiments we used a wider range of word lengths and lexical frequencies and binned the trials accordingly. Lexical frequency is a measure of how often a word occurs in large corpora of text, and correlates with familiarity and ease of recognition.

Fifth and finally, does a serial bottleneck constrain performance in any task as long as the stimuli are properly masked? In other words, is the deficit in the dual-task condition for semantic tasks due to the masking itself? We addressed that question in Experiment 3, using a color-detection task with the mask timing set to constrain accuracy in the same way as it did for the lexical and semantic judgments. In our previously published color detection experiments (White et al., 2018), the time between the words and the masks was matched to the semantic categorization condition, and was not set to the single-task threshold for color detection. The inter-stimulus interval (ISI) may therefore have been long enough to allow serial switching of attention to detect color in both words within one trial. Experiment 3 rectifies that concern.

To preview the results: performance in the semantic categorization and lexical decision tasks consistently ruled out the two standard parallel models and supported the all-or-none serial model. In contrast, the color-detection task supported a parallel model and was inconsistent with the all-or-none serial model, despite the strong masking. In the Discussion we consider several challenges to our interpretation of the data, including one related to the necessity of conscious awareness (Snell & Grainger, 2019a).

## Methods

### Experiment 1

#### Subjects

Ten volunteers (six female, ages 20–34 years, mean = 23.1 years) with normal or corrected-to-normal visual acuity participated in exchange for fixed monetary payment. Each subject gave informed consent in accordance with the Declaration of Helsinki and the University of Washington Institutional Review Board. All subjects were right-handed, naïve as to the purposes of the experiment, and had learned English as their first language. On the composite TOWRE-II Test of Word Reading Efficiency (Torgesen, Rashotte, & Wagner, 1999), all scored near or above the norm of 100 (M = 114, SEM = 4).

The sample size was chosen in advance of data collection on the basis of previous experiments with similar design (White et al., 2018). A power analysis suggested that in order to distinguish fixed-capacity parallel and all-or-none serial models with 95% power, on the basis of dual-task deficits and stimulus processing tradeoffs, we need at least 6 participants. We rounded that up to 10, to be conservative and consistent with our prior experiments.

#### Stimuli

We used custom MATLAB software (MathWorks, Natick, MA, USA) and the Psychophysics Toolbox (Brainard, 1997; Pelli, 1997) to present stimuli on a linearized CRT monitor (1,024 × 640 pixels; 120 Hz refresh rate; maximum luminance 90 cd/m^2^). The stimuli consisted of: a medium gray background (47 cd/m^2^), a small black fixation cross with dimensions 0.25 × 0.25 degrees of visual angle (°); and black letter strings in Courier font (28 pt; 4 cd/m^2^). The words were drawn from two semantic categories (“non-living” and “living”), each with 190 English nouns (available in the public repository for this study). Lexical frequency ranged from 0.06 to 539 per million with a median of 7.4 per million, according to the Clearpond database (Marian, Bartolotti, Chabal, & Shook, 2012). The words ranged from four to six characters in length, subtending 2.6–4.4° in width, and 0.6–1.1° in height. In addition, we used two types of post-masks: (a) strings of six random consonants, also black; (b) phase-scrambled images of consonant strings. Each phase-scrambled image was created by computing the Fourier transform of an image of consonants, replacing the phases with random values, and reverse transforming. The two mask types were thus matched in size (4.1–4.4° in width; 0.95–1.1° in height), root-mean-square luminance contrast, and spatial frequency content, but the phase-scrambled images contained no letters.

#### Trial sequence

**Fig. 1:**
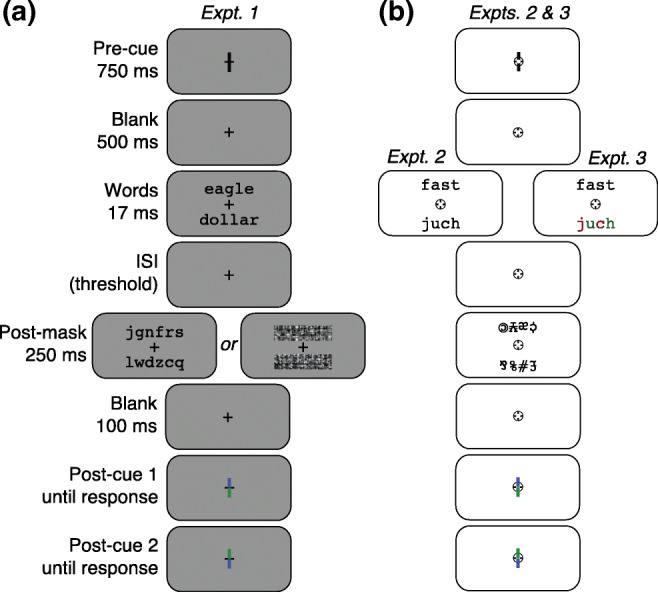
Example dual-task trial sequences. **a** Experiment 1, semantic categorization task. On single-task trials, the pre-cue was colored to direct attention to one side, and only that side was post-cued. A 25-ms click occurred 500 ms after the onset of post-cue 1, and again 300 ms after post-cue 2. Responses were not accepted before the clicks. Feedback beeps were played immediately after the last response on each trial. Not shown is the 1,000-ms inter-trial interval containing only the fixation mark. **b** Experiment 2 (lexical decision task) and Experiment 3 (color-detection task). The stimuli in these two experiments were identical except that in Experiment 3 half the letter strings were color targets: alternating red and green letters (as in “juch” in the rightmost panel). The trial sequence timing was identical in all three experiments, except for the word-mask ISIs. The mean ISIs in were: 36 and 15 ms for letter and phase-scrambled masks in Experiment 1, respectively; 61 ms in Experiment 2; and 31 ms in Experiment 3. The background was middle gray in Experiment 1 to allow for the phase-scrambled masks

As illustrated in Fig. 1a, each trial began with a 1,000-ms pre-cue: two vertical lines 0.15° long, one above and one below fixation, each with one end 0.05° from the center of the fixation mark. On dual-task trials, both pre-cue lines were black. On single-task trials, one was blue and one was green. Half the subjects were assigned to the blue cue, and half to the green. The line with the assigned color indicated the side (top or bottom) that would be post-cued on single-task trials. After a 500-ms blank interval containing only the fixation cross, the two words were flashed for 17 ms. The words were centered at 1.1° directly above and below fixation. Each word was equally likely to be drawn from either of the two semantic categories (living and non-living), independent of each other. The only constraints were that the words on the two sides could not be identical, and neither word could have appeared in the previous trial.

After the words was an ISI containing only the fixation mark, with duration set to the subject’s 80% correct single-task threshold. Table 1 lists the mean threshold ISIs used in each experiment. Details on how thresholds were determined are described in the *Procedure* section below. After the ISI, the two post-masks were presented for 250 ms, centered at the same locations as the preceding words. The mask type (consonants or phase-scrambled) varied randomly across trials, but both masks on each trial were of the same type. After another 100-ms blank interval, a post-cue appeared: two lines like the pre-cue lines, one green and one blue. After a 500-ms delay, a 25-ms click was played, which prompted the subject to press a key to report the category of the word on the side indicated by the post-cue line in their assigned color (blue or green). Key-presses before the click were not recorded.

**Table 1:** Inter-stimulus intervals (ISIs) between the words and the masks in each experiment. These ISIs were set to achieve 80–90% correct in the single-task conditions. The second column is the mean ISI across subjects. The third column indicates the range across subjects, computed by first taking the mean ISI across trials for each subject

Experiment	Mean ISI (ms)	Range (ms)
1: Semantic categorization (Consonant masks)	35	17–49
1: Semantic categorization (Phase-scrambled masks)	15	4–31
2: Lexical decision	61	33–92
3: Color detection	31	17–51

The task was semantic categorization: to report whether the post-cued word was a living thing or a non-living thing, along with confidence in the judgment. The subject pressed one of four keys with their left hand (*a*, *s*, *d* or *f*) when the post-cue pointed to the top side, or one of four keys with their right hand (*m*, *<*, *>*, or *?*) when the post-cue pointed to the bottom side. With each hand, the left-most key indicated “sure non-living” and the right-most key indicated “sure living.” The middle two keys indicated “guess non-living” and “guess living,” respectively, for when confidence was lower.

On *single-task* trials, the post-cue matched the pre-cue, prompting the subject to judge the category of the one attended word. As soon as the subject pressed a key, a 100-ms feedback tone was played: high pitch (600 Hz) if the response was correct, or low pitch (180 Hz) if the response was incorrect. Feedback was determined only by the reported category and not the confidence level. Then after a 1,000-ms inter-trial interval (ITI), the next trial began.

On *dual-task* trials, the subject had to judge the words on both sides, in a random order. Importantly, the categories of the two words were independent, so the correct answer for one side did not predict the correct answer for the other. After the post-mask, the post-cue pointed to one side, and the subject pressed one key. Then the post-cue reversed to point to the other side, and 300 ms later another click prompted the second response. After that, *two* feedback tones were played: one for the first response and another for the second response. Then came the ITI and the next trial.

#### Eye-tracking

We monitored the right eye’s gaze position with an Eyelink 1000 eye-tracker (SR Research). Fixation was established during the ITI at the start of each trial. The trial only advanced if the estimated gaze position was within 1.5° horizontally and 2° vertically of the fixation cross for at least 200 ms. We allowed more vertical tolerance to accommodate drifts due to pupil size changes. The gaze position averaged over the next ten samples was defined as the current trial’s fixation position. A fixation break was then defined as a deviation of gaze position more than 1° horizontally or 1.25° vertically from that fixation position. If a fixation break occurred between the pre-cue offset and post-mask offset, the trial was immediately terminated. The subject had to press a button to continue the next trial. Terminated trials were repeated at the end of the block, unless fewer than three trials remained. As described in the *Analysis* section below, we also detected fixation breaks greater than 1° vertically in offline analysis of the eye traces, and excluded those trials as well.

#### Procedure

Completing the experiment required seven to ten sessions each lasting one hour. In sessions 1–2 the subjects received instructions, read the list of words used in the experiment, practiced the task, and then ran a staircase procedure to estimate their ISI thresholds for both types of post-masks. The staircase was run in blocks of 20 trials, alternating between the single-task top condition and the single-task bottom condition (no dual-task trials in the staircase). During each run, the word-mask ISI in units of log_10_(seconds) was adjusted by a weighted 1-up/1-down staircase procedures controlled by the Palamedes toolbox (Prins & Kingdom, 2009). The step size down was always one-third of the step size up, which makes the staircase converge on the 75% correct threshold. Two staircases were randomly interleaved across trials, and blocks continued until both staircases had reversed direction ten times, and the threshold ISI was the mean value across all reversals. This whole procedure was run twice for both mask types tested separately in a random order, and threshold estimates were averaged across runs.

During the main experimental blocks (20 trials each), both mask types were randomly interleaved across trials, but the attention condition was blocked. Blocks were run in sets of four: two dual-task, one single-task top, and one single-task bottom, in a random order. Testing sessions continued until each subject had completed a total of 96 blocks (1,920 trials, half of which were dual-task). During each session, for each mask type, the ISI was constant across all conditions (dual-task and single-task).

The ISIs were initially set to the staircase threshold estimates but adjusted from session to session as necessary to keep single-task accuracy between 70% and 90% correct. Any run of four to 12 blocks with an ISI that was either too high (accuracy >90% correct) or too low (accuracy <70% correct) was discarded and re-run. This applied to 12 blocks for three subjects, and four blocks for one other.

Averaging across trials for each subject, the ISIs ranged from 17–49 ms (mean = 35 ± 3 ms) for consonant masks, and 4–31 ms (mean = 15 ± 2 ms) for the phase-scrambled masks. For all subjects, the ISIs were lower for the phase-scrambled masks than the consonant masks (mean difference = 20 ± 2 ms).

Finally, after the main experimental trials were finished, each subject ran 16 blocks of an “easy” condition with 400 ms ISI for both mask types. We used these easy blocks to assess accuracy when the masks were ineffective.

### Experiment 2

#### Subjects

Ten volunteers participated (three female, mean age 25.6 years, ranging from 19 to 36 years). As in Experiment 1, all had normal or corrected-to-normal visual acuity, gave informed consent, and participated in exchange for fixed monetary payment. Two had also participated in Experiment 1. With the exception of one left-handed author (AW), all subjects were right-handed and naïve as to the purposes of the experiment. With the exception of one bilingual speaker of Urdu, all had learned English as their first language. All scored above the norm of 100 on the TOWRE-II reading test (M = 112, SEM = 3).

#### Stimuli and procedure

All stimuli and procedures were identical to Experiment 1 except as described here. The display background was white (90 cd/m^2^), and all characters were black (4 cd/m^2^). In an effort to make fixation easier, the fixation mark was more complex: a black cross 0.3° wide, with a 0.1° white dot at its center, and a thin black ring around it (0.3° diameter).

The stimulus set was composed of 702 real English words and 702 pronounceable pseudowords (available in the public repository for this study). Both categories were divided equally into strings of three, four and five letters long. We used a lower range of lengths here than in Experiment 1 to test the hypothesis that two very short words could be recognized in parallel. The real words came from all syntactic categories, ranging in lexical frequency from 3.4 to 873 occurrences per million. The four- and five-letter pseudowords had matched constrained trigram statistics to real words, and the three-letter pseudowords had matched constrained bigrams (Medler & Binder, 2005). Therefore, the pseudowords were pronounceable, with phonemic characteristics similar to real words. The masks were strings of non-letter characters drawn randomly from the set: ¢, ß, æ, ¥, ©, £, @, #, %, &. We generated a set of 702 unique masks with the same length distribution as the words. The masks were presented upside-down.

On each trial, two letter strings were presented simultaneously, one above and one below fixation, centered at 1.5° eccentricity. We increased the eccentricity in this experiment (compared to 1.1° in Experiment 1) to make it easier to process the two stimuli independently and avoid looking directly at either one. The two strings were the same length, and each had an independent 50% chance of being a real word. The masks were matched in length to the preceding letter strings, and presented upside down at the same locations.

During each trial, a fixation break was defined as a deviation of the right eye’s gaze position more than 1° horizontally or 1° vertically. This criterion was made more conservative than in Experiment 1 out of an abundance of caution, to ensure that all fixation breaks were detected.

The task was lexical decision: to report whether the post-cued letter string was a pseudoword or a real word. As in Experiment 1, the subjects pressed one of four keys for each post-cued side, to report the stimulus category and their level of confidence (from “sure pseudoword” to “sure real word”).

Given that there was only one mask type, we only had to estimate one ISI threshold for each subject, using the same staircase procedure. The across-trial average ISIs ranged from 33 to 92 ms (mean = 61 ± 7 ms). The fact that these ISI thresholds were longer than in Experiment 1 could be explained by the greater retinal eccentricity (1.5° vs. 1.1°), which made the stimuli somewhat more difficult to perceive.

Each subject completed a total of 60 blocks (1200 trials), over four to five 1-h sessions. No blocks had to be excluded and re-run due to the difficulty level being out of range. Unlike Experiment 1, there was no “easy” condition with a long ISI.

### Experiment 3

#### Subjects

Ten volunteers participated (three female, mean age 25.4 years, ranging from 19 to 35 years). As in Experiments 1 and 2, all had normal or corrected-to-normal visual acuity, gave informed consent, and participated in exchange for fixed monetary payment. Two had also participated in Experiment 2, and two were left-handed. With the exception of one author (AW), all subjects were naïve as to the purposes of the experiment. With the exception of the same bilingual speaker of Urdu from Experiment 2, all had learned English as their first language. All participants were screened for normal color vision using Ishihara color plates.

#### Stimuli and procedure

All stimuli and procedures were identical to Experiment 2 except as described here. We used the same set of real words and pseudowords as in Experiment 2, except their luminance was set to 17% of the maximum (18.2 cd/m^2^; 83% Weber contrast). On each trial, each letter string had an independent 50% chance of being a color target: its letters alternated in color between red and green (with the first color randomized). The non-target letter strings were all dark gray, and roughly equiluminant with the reds and greens.

The task was color detection: to report whether the post-cued letter string was colored or gray. As in Experiment 2, the subjects pressed one of four keys for each post-cued side, to make a rating from “sure gray” to “sure colored”.

Adjusting the stimulus difficulty for each subject proceeded in two stages: first, we adjusted the saturations of the red and green colors to be roughly equally salient and to allow for >90% correct detection with 300 ms ISI. To adjust the saturations while keeping luminance roughly constant, we used the measured luminance outputs of each monitor gun. Starting with the baseline dark gray, we incremented the intensity of one gun (green or red) and decremented the other two by however much was necessary to keep the total luminance constant. This allowed for 132 red colors and 20 green colors, varying from gray to the maximum saturation available (corresponding to when the other two guns were at 0).

We express those saturation levels as proportions of the maximum while maintaining constant luminance. The mean (± SEM) red saturation proportion was 0.68 ± 0.05, and the mean green saturation proportion was 0.94 ± 0.03. One participant (S3) struggled to perform the task even with maximum saturations, so for that participant the duration of the letter strings was increased from 17 ms to 25 ms.

Then, with the color levels fixed, we adjusted the ISI to threshold, to achieve roughly 80% correct performance in the single-task condition. This was done by hand in practice blocks, rather than with a full staircase procedure. Across subjects, the threshold ISIs ranged from 17 to 51 ms (mean = 31 ± 3 ms).

During each trial, a fixation break was defined as a deviation of the right eye’s gaze position more than 1° horizontally or 1.25° vertically.

As in Experiment 1, we included some “easy” blocks with a long ISI (300 ms). To ensure that we set the color saturation levels appropriately, eight easy blocks were run before any of the main experimental blocks. Twelve more easy blocks were run at the end of the last session. In total, each subject completed 60 main experimental blocks (1200 trials) and 20 easy blocks (400 trials), in five to nine sessions. No blocks had to be excluded and re-run due to the difficulty level being out of range.

### Analysis

#### Behavioral accuracy

In all three experiments, the subject’s task was to report which of two categories a letter string belonged to, along with a confidence rating. To analyze the subjects’ sensitivity, we re-labelled one category as “targets” and the other “non-targets.” A “target-present” trial was then defined as a trial in which the post-cued stimulus was from the target category. We then re-coded each response as a 1–4 rating from “sure target absent” to “sure target present.” The target categories in Experiments 1, 2, and 3 are: “living” words, real words, and colored letter strings, respectively.

As a bias-free measure of accuracy in each condition, we computed the area under the receiver operating characteristic (ROC) curve, A_g_ (Pollack & Hsieh, 1969). The ROC plots hit rates (HR) as a function of false alarm rates (FR). To compute these rates from the subjects’ response ratings, we varied an index *i* from 0 to 4. At each index level we coded responses greater than *i* as “yes” responses. For each value of *i*, HR(*i*) is the proportion of “yes” responses on target-present trials and FR(*i*) is the proportion of “yes” responses on target-absent trials. For instance, when *i* = 3, only response ratings of 4 (highest confidence) on target-present trials are considered hits, and only response ratings of 4 on target-absent trials are considered false alarms. The five pairs of HR(i) and FR(i) trace out a curve, the area under which (A_g_) is a measure of accuracy. A_g_ ranges from 0.5 (chance) to 1.0 (perfect). One can think of A_g_ as an unbiased estimate of proportion correct.

#### Gaze fixation

During the experiments, fixation breaks were detected online and those trials were immediately terminated (and therefore excluded from the analysis). To be sure that we included no trials in which subjects may have looked directly at a word, we also analyzed the eye traces offline. First, for each trial in a block, we computed the median gaze position (across measurement samples) in the 300 ms before the pre-cue onset (excluding intervals with blinks). Then we defined the “central gaze position” for the block as the across-trial median of those initial gaze positions. This analysis corrects for any error in the eye-tracker calibration by assuming that subjects were fixating correctly in the interval before the pre-cue, when only the fixation mark was visible.

Then, for each trial, we analyzed gaze positions in the interval between the onset of the words and the offset of the post-masks. We defined an “offline fixation break” as a deviation that was more than 3° horizontally or 1° vertically from the central gaze position and that lasted more than 30 ms. In the analysis, we excluded all trials with offline fixation breaks. That led to an average loss of 4.9 ± 1.2% of the data in Experiment 1, 2.6 ± 0.9% in Experiment 2, and 3.5 ± 1.8% in Experiment 3.

#### Bootstrapping

Throughout the text we report bootstrapped 95% confidence intervals (CIs) for average measurements. To compute these, we generated a distribution of 5,000 resampled means. Each of those is the mean of ten values sampled with replacement from the original set of ten subjects’ means. The CI is the range from the 2.5th to 97.5th percentile of the distribution of resampled means, with an “accelerated” bias correction (Efron, 1987).

## Results

### Dual-task deficits and attention operating characteristics

In this paradigm, the primary evidence for a processing capacity limit is a dual-task deficit: lower accuracy compared to the single-task condition. Table 2 lists the mean (and SEM) accuracies in each of condition of the three experiments, collapsing across top and bottom sides. Accuracy is in units of Area under the ROC curve (A_g_). All three experiments had significant dual-task deficits (p < 0.01, CI excludes 0), but they were roughly three times larger in the semantic and lexical tasks than in the color-detection task. In Experiment 1 (semantic categorization), the dual-task deficit was slightly higher with masks made of constants than phase-scrambled consonants, but not significantly so (mean difference in deficit = 0.02 ± 0.01; t(9)=1.94, p=0.084; CI = [-0.002 0.036]). Experiments 2 (lexical decision) and 3 (color detection) used very similar stimuli, so we directly compared them. The dual-task deficit in Experiment 2 (0.21) was significantly larger than in Experiment 3 (0.06): t(18)=7.15, p<10^-5^, CI of difference = [0.11 0.18].

**Table 2:** Mean accuracies (in units of A_g_) and dual-task deficits in the three experiments, with Experiment 1 (semantic categorization task) divided by the two mask types (N = 10)

Experiment	Single-task	Dual-task	Deficit
1 (Semantic; consonants)	0.88 (0.01)	0.69 (0.01)	0.19 (0.02) [0.17 0.23]
1 (Semantic; phase-scrambled)	0.89 (0.01)	0.72 (0.01)	0.17 (0.01) [0.15 0.20]
2 (Lexical decision)	0.88 (0.01)	0.67 (0.02)	0.21 (0.01) [0.18 0.23]
3 (Color detection)	0.82 (0.01)	0.76 (0.01)	0.06 (0.02) [0.03 0.09]

Numbers in parentheses are standard errors of the mean, and numbers in brackets are 95% bootstrapped confidence intervals (CIs)

We also examined any differences in accuracy between the first and second responses in dual-task trials. In all three experiments, the mean differences (second – first) were small and not statistically significant: Experiment 1: -0.011 ± 0.009 A_g_ (CI = [-0.026 0.006]); Experiment 2: -0.026 ± 0.012 (CI = [-0.049 0.004]); Experiment 3: 0.008 ± 0.009 (CI = [-0.009 0.025]). Therefore, the large dual-task deficits in Experiments 1 and 2 cannot be explained by a failure to remember both words.

**Fig. 2:**
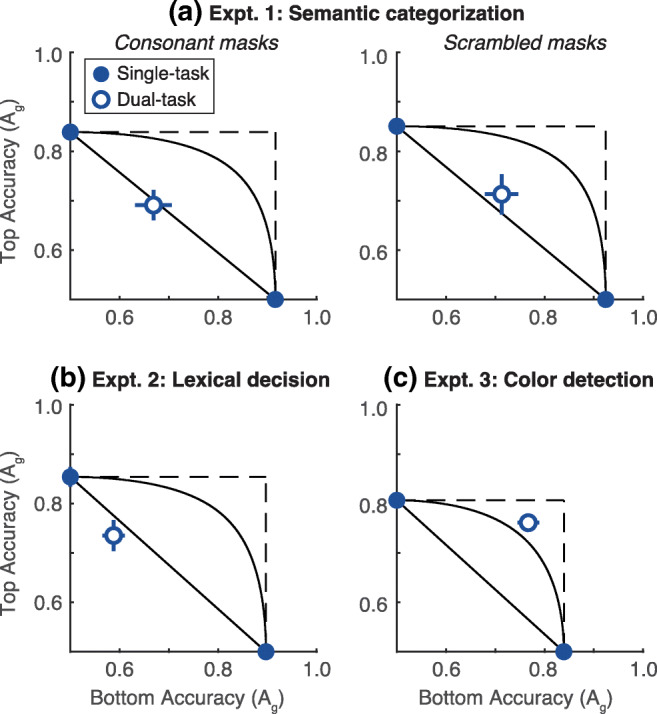
Mean attention operating characteristics in all three experiments. Solid points pinned to the axes are single-task accuracy levels (in units of area under the ROC curve, A_g_). Open points are dual-task accuracy levels. Error bars show ±1 SEM (N = 10). In Experiments 1 (**panel a**) and 2 (**panel b**), dual-task accuracy is closest to the all-or-none serial model’s prediction (diagonal line). In Experiment 2 (**panel c**), it is closest to the fixed-capacity parallel model’s curved prediction. Individual subject AOCs are plotted in the Supplementary Material.

To compare the dual-task deficits to model predictions, we plot our data on attention operating characteristics (AOCs; Sperling & Melchner, 1978). The mean AOCs for each experiment are in Fig. 2: accuracy for words above fixation is plotted against accuracy for words below fixation. The single-task conditions are pinned to their respective axes. The accuracy levels in the dual-task condition form a single point (open circle) in that 2-D space. We compared that point to the predictions of three specific models of capacity limits (Bonnel & Prinzmetal, 1998; Scharff, Palmer, & Moore, 2011; Shaw, 1980; Sperling & Melchner, 1978; White et al., 2018):

1. *Unlimited-capacity parallel processing*: Two stimuli can be fully processed simultaneously just as well as one stimulus, so there is no dual-task deficit. In the AOC, this model predicts that the dual-task point falls at the intersection of the dashed lines.
2. *Fixed-capacity parallel processing*: The perceptual system extracts a fixed amount of information from the whole display per unit time. Therefore, processing resources must be shared between both stimuli in the dual-task condition, which lowers sensitivity. As the proportion of resources given to the right stimulus increases from 0 to 1, this model traces out the black curve in the AOC plot.
3. *All-or-none serial processing*: Only one stimulus can be processed per trial, with equal sensitivity as in the single-task condition. The subject does not have time to even start processing the other stimulus and therefore must guess when asked about it. As the proportion *v* of trials in which the right side is processed increases from 0 to 1, this model traces out the diagonal black line in the AOC plot.

More information, including how the prediction curves were calculated, is in the Appendix. In addition, the Supplementary Material contains AOCs for individual subjects in all three experiments (Figures S1–S3).

#### Experiment 1

As shown in Fig. 2a, mean accuracy for both mask types in the semantic categorization task was best predicted by the all-or-none serial model. For each subject individually (plotted in the Supplement, Figure S1), we computed the Euclidean distance of the dual-task point from the nearest point on the diagonal serial model prediction line, and from the nearest point on the fixed-capacity parallel model’s prediction curve. Points below the predictions were assigned negative values. For consonant masks, the mean distance from the serial model’s prediction was -0.01 ± 0.02, not significantly different from 0 (t(9) = -0.60, p = 0.56, CI = [-0.04, 0.01]). The mean distance from the fixed-capacity parallel prediction was significantly negative: -0.09 ± 0.02 (t(9) = 4.44, p = 0.0016, CI = [-0.14, -0.06]).

For the phase-scrambled masks, the all-or-none serial model also fit best. The mean distance from the serial model line was 0.03 ± 0.02 (t(9)=1.48, p = 0.17, CI = [-0.01, 0.061]). That distance was significantly greater than for the consonant masks: mean difference = 0.04, ± 0.02; t(9) = 2.38, p = 0.041, CI = [0.01, 0.06]. For phase-scrambled masks, the mean distance from the fixed-capacity parallel curve was = -0.07 ± 0.012, t(9) = 6.31, p = 0.0001, CI = [-0.10, -0.05].

Therefore, for both mask types, dual-task accuracy was significantly worse than predicted by the fixed-capacity parallel model and near the prediction of the all-or-none serial model. The serial model assumes that subjects could semantically categorize one of the two words (with the same probability correct as in the single-task condition), but had to guess about the other. These results are similar to what we have reported before (White et al., 2018, 2019), but here generalized to positions above and below fixation, and to masks that do not contain letters.

#### Experiment 2

As shown in Fig. 2b, performance in the lexical decision task was also worse than predicted by either parallel model and consistent with the all-or-none serial model. Mean dual-task accuracy was slightly (but not significantly) below the serial model’s prediction. The mean distance was = -0.03 ± 0.02 (t(9) = 1.49, p = 0.17, CI = [-0.07, 0.01]). Accuracy was significantly below the fixed-capacity parallel model: mean distance = -0.11, SEM ± 0.02; t(9) = 5.99, p = 0.0002, CI = [-0.15, -0.08].

Therefore, we can rule out the fixed-capacity parallel model even when the task requires lexical access but doesn’t require making decisions about the semantic meaning of the words.

#### Experiment 3

As shown in Fig. 2c, performance in the color-detection task was most consistent with the fixed-capacity parallel model, unlike in the word-recognition tasks in Experiments 1 and 2. Dual-task accuracy was significantly above the serial model line: mean distance = 0.14 ± 0.02; t(9) = 7.14, p = 0.0001, CI = [0.09, 0.17]. Compared to the fixed-capacity parallel model’s prediction, dual-task accuracy was slightly but not significantly better: mean distance = 0.03 ± 0.02; t(9) = 1.55, p = 0.155, CI = [-0.01, 0.06].

In a previous color detection experiment, we found that dual-task accuracy was significantly above the fixed-capacity prediction, near the unlimited-capacity prediction (White et al., 2018). However, in that experiment the word-mask ISI was not set to limit performance in color detection. Therefore, the masking in that previous experiment might have been less effective than it was for the semantic task.

Here, we ensured that the masks were effective for the color task and reduced the ISI to limit single-task performance just as for the semantic and lexical decision tasks. To demonstrate that, we also included blocks with long ISIs in Experiments 1 and 3 (400 and 300 ms ISIs, respectively). In the single-task conditions, mean accuracy with the long ISI was 0.99 ± 0.003 in Experiment 1 and 0.95 ± 0.01 in Experiment 3. Therefore, the task was easy when given sufficient processing time. When the ISI was reduced to threshold, accuracy fell greatly: by 0.11 ± 0.01 A_g_ units in Experiment 1, and by 0.13 ± 0.02 A_g_ units in Experiment 3. Both of those effects of shortening the ISI were significant (both t(9)>8, p < 10^-5^), and did not differ significantly from each other (t(18) = 1.11, p = 0.28; CI = [-0.01, 0.05]).

Dual-task accuracy was also high with long ISIs: 0.95 ± 0.01 in Experiment 1 and 0.92 ± 0.02 in Experiment 3. Therefore, both words could be fully processed on most trials if there was sufficient processing time before the masks appeared, even for the semantic categorization task. The effect of reducing the ISI to threshold in the dual-task condition was 0.09 A_g_ units greater in Experiment 1 (semantic categorization) than in Experiment 3 (color detection): t(18) = 4.62, p = 0.0002; CI = [0.05, 0.13]. This is because within the amount of time allowed by threshold ISIs, only one word’s meaning can be fully recognized, but the color of both words can be processed in parallel (as demonstrated in the AOCs in Fig. 2).

Therefore, the effects of masking *per se* cannot explain the large dual-task deficits for the semantic and lexical judgments.

### Effects of string length and lexical frequency

We next investigated whether the capacity limit in dual-task performance depends on how long the words are, and how common they are in the lexicon. Perhaps two short, very common words could be processed in parallel.

In our stimulus set, lexical frequency (measured as occurrences/million) and word length were negatively correlated. More common words tend to be shorter. Therefore, for each experiment we split the trials into two sets defined jointly by the frequency and length of the words presented: (1) low-frequency long words, and (2) high-frequency short words.

“Low-frequency” words were in the bottom 33% of all the words used in the experiment: 0.06–3.4 per million in Experiment 1, and 3.4–12.2 per million in Experiments 2 and 3. “High-frequency” words were in the upper 33%: 14.5–539 per million in Experiment 1, and 50.1–872 in Experiments 2–3. “Long” words were the longest used in each experiment: six letters in Experiment 1, and five letters in Experiments 2–3. “Short” words were the shortest used: four letters in Experiment 1, and three letters in Experiments 2–3.

**Fig. 3:**
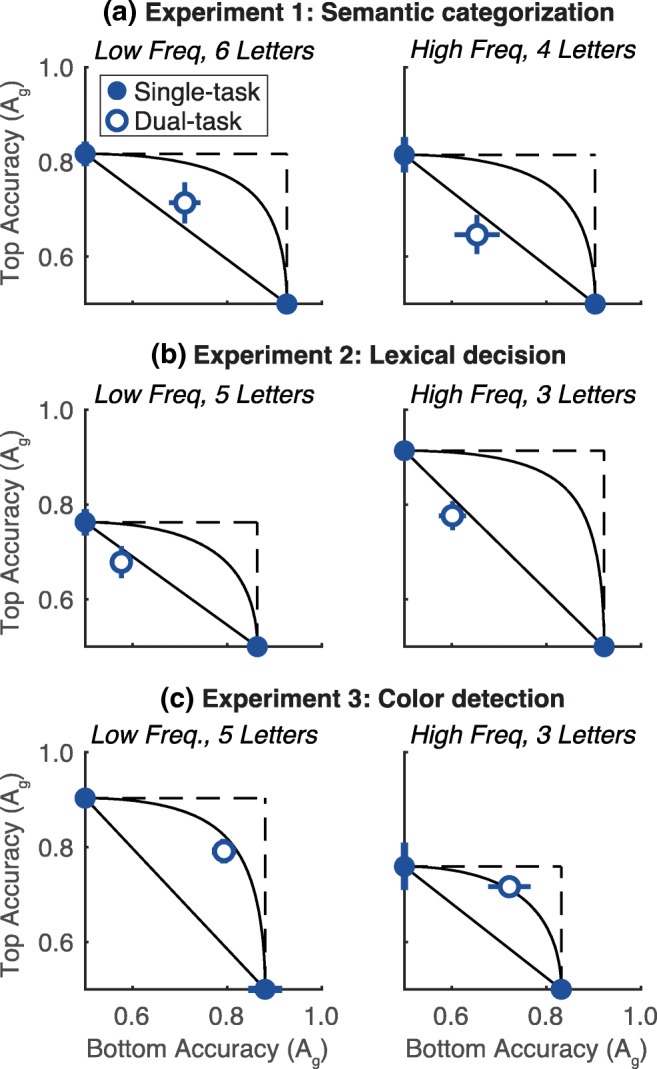
Attention operating characteristics for low-frequency long words (**left column**) and high-frequency short words (**right column**) in each of the three experiments (rows **a**, **b,** and **c**). Format as in Fig. 2

We constructed AOCs for both sets of trials (low-frequency long words, and high-frequency short words). The means are plotted in Fig. 3. If short and common words can be processed in parallel, we would predict dual-task accuracy to rise above the all-or-none serial model’s prediction for the second subset of trials (right column of Fig. 3). That did not occur in Experiments 1 or 2.

In Experiment 1, single-task accuracy was similar in the two sets of trials (mean difference = -0.01 ± 0.02), but dual-task accuracy was actually better for the low-frequency long words (mean difference = 0.06 ± 0.02, t(9) = 3.19, p = 0.01, CI = [0.03, 0.10]). This result was driven by the counterintuitive effect of word length in Experiment 1 (see discussion below and Fig. S4). For the high-frequency short words (top right panel in Fig. 3), dual-task accuracy was significantly below the fixed-capacity parallel prediction (mean distance = -0.11 ± 0.03, t(9) = 3.08, p = 0.013, CI = [-0.17, -0.04]).

In Experiment 2 (lexical decision), single-task accuracy was much higher for the high-frequency short words than low-frequency long words (mean difference = 0.11 ± 0.01; t(9) = 7.64, p < 0.0001, CI = [0.08, 0.13]). That was also true in the dual-task condition (mean difference = 0.06 ± 0.01, t(9) = 5.34, p = 0.0005, CI = [0.04, 0.09]. Nonetheless, even for the high-frequency short words (middle right panel in Fig. 3), dual-task accuracy fell significantly below the fixed-capacity parallel prediction (mean distance = -0.13 ± 0.02, t(9)=5.97, p = 0.0002, CI = [-0.17, -0.08]).

In Experiment 3 (color detection), single-task accuracy was higher for the low-frequency long words (the opposite pattern as in Experiment 2; mean difference = -0.10 ± 0.03; t(9) = 3.06, p = 0.014, CI = [-0.17, -0.05]). That effect was driven by length: longer words make the detection task easier, because there are more colored letters in the targets. For both sets of trials in Experiment 3, dual-task accuracy was near the fixed-capacity parallel prediction, and significantly above the all-or-none serial model prediction (both p < 0.01), consistent with the overall analysis in Fig. 2.

In the Supplementary Material we also report how accuracy was affected by lexical frequency and length separately (Fig. S4). The effects of length were variable across experiments. Unlike in Experiment 2, accuracy in Experiment 1 was better for longer words, for reasons we cannot fully explain. The effect of lexical frequency was consistent across both Experiments 1 and 2: words with higher lexical frequencies were easier to recognize in (bottom row of Fig. S4).

More importantly for the question at hand: for each manipulation (e.g., increasing lexical frequency) that increased single-task accuracy, dual-task accuracy increased by the same amount or less. This pattern was most striking in Experiments 1 and 2: the effect of lexical frequency was significantly smaller in the dual-task condition than in the single-task condition. As a result, the relative dual-task deficit was *larger* for high- than low-frequency words. One way to interpret this finding is that in the dual-task condition, only half the words get to the stage of processing at which lexical frequency influences the recognition process. In other words, the serial bottleneck lies prior to the stage at which common words are recognized as familiar (see *Discussion*).

In summary, we found no evidence that two words can be recognized in parallel if they are short and common. The serial model consistently held for the semantic and lexical tasks, while the fixed-capacity parallel model consistently held for the color-detection task.

### Stimulus processing tradeoffs

The all-or-none serial model assumes that in the dual-task condition, the subject fully processes the top stimulus on some trials and the bottom stimulus on others, but never both. Therefore, there’s a trial-by-trial *tradeoff* between the two stimuli. The model accordingly makes an additional prediction: accuracy for each side should be lower when the response to the other side was *correct* than incorrect (Braun & Julesz, 1998; Lee, Koch, & Braun, 1999; Sperling & Melchner, 1978; White et al., 2018). For instance, if the response to the top side is correct, the top side was probably processed and therefore the bottom side was probably not, so the response to the bottom side is less likely to be correct.

**Fig. 4:**
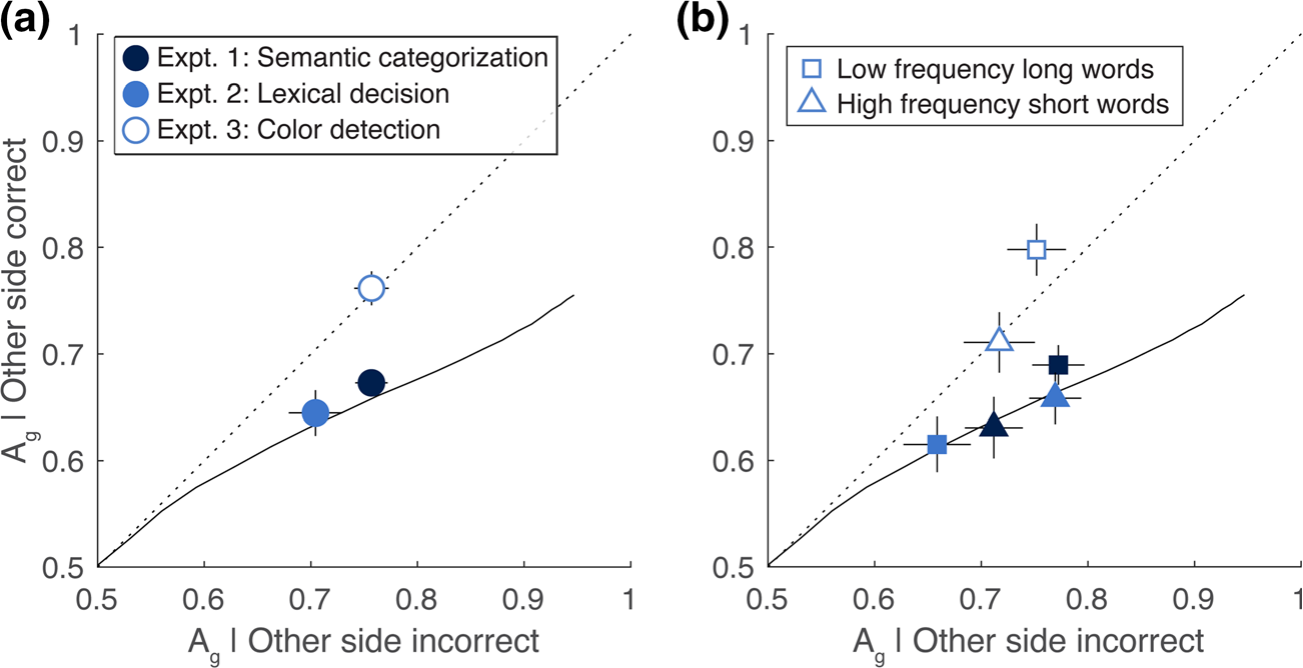
Stimulus processing tradeoffs in the dual-task conditions of all three experiments. (**A**) Analysis including all dual-task trials. The dotted diagonal line is the prediction of the fixed-capacity parallel model. The curved solid line is the prediction of the all-or-none serial model, generated by varying single-task discriminability. **(B)** The same analysis, but for the two subsets of trials used in Fig. 3: low-frequency long words and high-frequency short words. Symbol colors indicate experiment as in panel A. Error bars show ±1 SEM (N = 10)

We tested that prediction by dividing all responses on dual-task trials into two sets: (1) the response to the other side was correct, and (2) the response to the other side was incorrect. Within each set we computed accuracy (A_g_). The means for all three experiments are shown in Fig. 4a: accuracy when the other side’s response was incorrect is on the horizontal axis, and accuracy when the other side’s response was correct is on the vertical axis. Points below the identity line indicate that there was a tradeoff (worse performance when the other side was correct). The curved black line in Fig. 4a is the prediction of the all-or-none serial model. We generated this prediction by simulating thousands of trials, varying discriminability to sweep out the curve. See the Appendix for details.

For Experiment 1 (semantic categorization), accuracy was significantly lower when the other side’s response was correct than incorrect: mean difference = 0.08 ± 0.02 (t(9) = 5.07, p = 0.0007, CI = [-0.11, -0.05]. Nine of ten subjects showed the effect in the predicted direction. That was also true in Experiment 2 (lexical decision): mean difference = -0.06 ± 0.02, t(9) = 3.04, p = 0.014, CI = [-0.09, -0.02]. Eight of ten subjects showed the effect in the predicted direction. In Experiment 3 (color detection), however, there was on average no effect of the other’s response’s accuracy: mean difference = 0.005 ± 0.02, t(9) = 0.24, p = 0.81, CI=[-0.03, 0.04]. The ten subjects were evenly split in terms of the sign of that effect.

We went one step further to analyze the stimulus processing tradeoffs for separate sets of words, as in Fig. 3: low-frequency long words and high-frequency short words. The results are in Fig. 4b. Squares are for low-frequency long words; triangles are for high-frequency short words. The data for Experiments 1 and 2 (semantic and lexical tasks in black and blue, respectively) roughly follow the prediction of the all-or-none serial model. The tradeoff effect was generally larger for conditions for higher overall accuracy. In contrast, data from both sets of trials in Experiment 3 (color detection) were on or (not significantly) above the diagonal identity line predicted by the fixed-capacity parallel model.

In the Supplementary Material we report an alternative analysis of this effect: across-trial correlations between the accuracies for the top word and the bottom word (Bonnel & Prinzmetal, 1998; Ernst, Palmer, & Boynton, 2012; Lee et al., 1999; Sperling & Melchner, 1978). The correlation results are consistent with the stimulus tradeoffs, but we prefer the latter analysis because it is less affected by criterion shifts and appears to be more reliable.

To summarize, in the semantic categorization and lexical decision experiments we found evidence of a stimulus processing tradeoff in the dual-task condition. If subjects correctly judged one side, they were less likely to correctly judge the other. Those effects were near the prediction of the all-or-none serial model, which also accounts for the large dual-task deficits. There was no such tradeoff between the two stimuli in the color detection experiment, for which the modest dual-task deficit was best accounted for by the fixed-capacity parallel model.

### Effects of congruency

In a wide range of visual tasks, the subject’s response to one stimulus is influenced by the identity of other stimuli nearby. For instance, in the classic “flanker effect” (Eriksen & Eriksen, 1974), subjects are instructed to discriminate a target that is flanked by irrelevant stimuli. Performance is better when the flankers correspond to the same category as the target (congruent stimuli) than when they correspond to the opposite category (incongruent stimuli). That effect of congruency is evidence that the subject did not completely filter out the irrelevant flankers.

We can also analyze effects of stimulus congruency in our data. They reveal how well our subjects selectively attended to just one word in the single-task condition, and how well they processed the two words independently of each other in the dual-task condition. Moreover, the existence of congruency effects is often taken as evidence of parallel processing. But as we argue below, serial models can also explain them.

We compared accuracy on “congruent” trials, when the stimuli on both sides belong to the same category, with accuracy on ‘incongruent’ trials, when the two stimuli belong to different categories. The relevant categories in each experiment are: (1) living and non-living things; (2) pseudowords and real words; and (3) gray letters and colored letters. Table 3 lists the mean congruency effects in each condition.

**Table 3:** Magnitudes of congruency effects on accuracy each experiment

	Single-task		Dual-task	
Expt.	Mean (SEM)	95% CI	Mean (SEM)	95% CI
1 (Semantic)	0.02 (0.01)	[0.01, 0.04]*	0.10 (0.04)	[0.04, 0.19]*
2 (Lexical)	0.01 (0.01)	[-0.01, 0.02]	0.04 (0.01)	[0.01, 0.06]*
3 (Color)	0.06 (0.01)	[0.05, 0.09]***	0.06 (0.02)	[0.01, 0.10]*

Effects are reported as the mean difference in accuracy (A_g_) between congruent and incongruent trials

* p < 0.05, *** p < 0.001

In Experiment 1 (semantic categorization), there was a small congruency effect (0.02 A_g_ units) in the single-task condition and a large effect (0.10) in the dual-task condition. In Experiment 2 (lexical decision), there was effectively no congruency effect in the single-task condition but a modest one (0.04) in the dual-task condition. Therefore, subjects in Experiments 1 and 2 efficiently attended to just one word in the single-task conditions, but when attending to both words they were not completely able to process and respond to them independently (especially in Experiment 1). Finally, in Experiment 3 (color detection), there was a congruency effect in both the dual-task and single-task conditions (0.06). These patterns were very similar when analyzing d’ instead of A_g_.

Some authors explain congruency effects with the concept of c*ross-talk:* interactions between the representations of the two stimuli while their task-relevant features are processed in parallel (Hübner & Lehle, 2007; Logan & Gordon, 2001; Mordkoff & Yantis, 1991; Navon & Miller, 1987). As the two representations rise towards a threshold for the correct decision, they facilitate each other if they’re of the same category (improving accuracy on congruent trials) and/or interfere with each other if they’re of opposite categories (causing errors on incongruent trials).

However, congruency effects can also be explained by *selection errors:* occasionally swapping information from the two sides. For instance, the subject may report what they saw on the top side when the bottom side is post-cued. This would cause errors on only incongruent trials, because mixing up the sides on congruent trials would have no effect (Lachter, Forster, & Ruthruff, 2004; Palmer & Moore, 2009; Yantis & Johnston, 1990).

The selection-error hypothesis is consistent with both serial and parallel models. Therefore, the all-or-none serial model that is supported by the AOCs and stimulus processing tradeoffs for Experiments 1 and 2 can also explain the dual-task congruency effects by assuming that on some proportion of trials, subjects do not perfectly follow the post-cue, and report what they saw on the wrong side. A related hypothesis is that the subjects are sometimes biased to report the same answer for both sides, especially when uncertain about one. Such a bias would cause more errors on incongruent trials than on congruent trials, although only one stimulus was fully processed on each trial.

The small congruency effect in the single-task condition of Experiment 2 could also be explained within the serial model by an occasional failure to attend selectively to the pre-cued side (Lachter et al., 2004; Palmer & Moore, 2009; Yantis & Johnston, 1990).

### Differences between top and bottom sides

In our previous experiments (White et al., 2018, 2019) we observed that semantic categorization accuracy was greater for words to right than left of fixation. In the present study we examined differences in accuracy between the positions above and below fixation, as shown in Table 4. In the single-task conditions of Experiments 1 and 2, there was a moderate advantage for the *bottom* side, consistent generally better visual performance in the lower visual field (Carrasco, Talgar, & Cameron, 2002). However, that effect disappeared or even reversed in the dual-task conditions, with significantly better dual-task performance for the *top* side in Experiment 2. The latter effect could be due to a strategy to start with the top side on dual-task trials, given that subjects couldn’t process both sides simultaneously, and the usual reading direction is top to bottom (Goodbourn & Holcombe, 2015; Holcombe, Nguyen, & Goodbourn, 2017; Ransley, 2018).

**Table 4:** Mean differences in accuracy (Ag) between the two sides: top-bottom

Experiment	Single-task	Dual-task
1 (Semantic)	-0.07 (0.01) [-0.11, -0.05]***	0.01 (0.07) [-0.13, 0.12]
2 (Lexical)	-0.04 (0.02) [-0.08, -0.01]*	0.15 (0.04) [0.09, 0.22]**
3 (Color)	-0.03 (0.02) [-0.08, 0.01]	-0.01 (0.03) [-0.05, 0.05]

Numbers in parentheses are SEMs, and numbers in brackets are 95% bootstrapped CIs

* p < 0.05; **p < 0.01; *** p < 0.001

In Experiment 3 there were no significant effects of side in either condition.

## Discussion

### Summary

Based on data from a range of stimulus and task conditions, we conclude that skilled readers cannot recognize two written words simultaneously. Specifically, we reject two standard parallel-processing models of accuracy in two tasks that require lexical access. The experiments presented here generalize our previously reported evidence for a serial bottleneck in word recognition (White et al., 2018) in several ways: (1) for lexical decision (distinguishing real words from pseudowords) as well as for semantic categorization (distinguishing “living” from “non-living” nouns); (2) for words directly above and below fixation, rather than in opposite hemifields; (3) for three types of post-masks (letter strings, phase-scrambled noise patches, and upside down characters); (4) for words of varying length and lexical frequency. Finally, we found again that judgments of text color benefit from parallel processing, even when the color itself was effectively masked.

For semantic and lexical judgments, dual-task accuracy was far below the predictions of two standard parallel models and in line with the all-or-none serial model (Fig. 2). This serial model assumes that the subject can recognize one word with the same level of accuracy as in the single-task condition, but cannot extract any relevant information from the second word and has to guess. This serial model was also supported by a trial-by-trial stimulus processing tradeoff between the two words. Accuracy for each side was higher when the other side was judged incorrectly than correctly (Fig. 3). However, in the color-detection task (Experiment 3), the AOC was consistent with fixed-capacity parallel processing, and there was no tradeoff.

### Masks and time limits on processing

The favored serial model is called “all-or-none” because it assumes that one word is processed fully, but the task-relevant attribute (e.g., semantic category) of the other word is not processed at all. A more general serial model may assume that both words are processed on each trial, but one after the other. The reason the all-or-none model fits our data is that there isn’t enough *time* to process more than one word. The masks replace the words after a delay that is calibrated to allow just enough time to recognize one word in the single-task condition. If participants could process two words in parallel in that same amount of time, then they would perform above chance for both words in the dual-task condition, but they do not.

Importantly, when we increased the time between the words and the masks to 400 ms, semantic categorization accuracy was at ceiling in the single-task condition and near 95% correct in the dual-task condition. Therefore, although the words were present for only 17 ms, our subjects *could* recognize them both, but only if given enough subsequent processing time.

The post-masks are critical to understanding our result. The masking need not be at the level of orthographic representations, because it also works for noise patches and upside-down non-letter characters. It seems that any high-contrast visual pattern can interrupt processing of the words if it follows them quickly enough.

Our interpretation that the masks interrupt processing may be too simplistic (Bridgeman, 2006; Enns & Di Lollo, 2000; Felsten & Wasserman, 1980; Holender, 1986). The masks might do something else that makes the visual word recognition system behave in an “unnatural” way that does not occur during natural reading. Even so, the serial result (large dual-tasks deficits and stimulus processing tradeoffs) is not an obligatory consequence of the masking. In the color-detection task (Experiment 3), we used the same masks as in Experiment 2, and they constrained single-task accuracy in a similar way: accuracy was high (~95% correct) with a long ISI, and fell to a threshold level (~82% correct) when the ISI was sufficiently reduced. Thus, color detection sensitivity is also sensitive to the amount of processing time allowed by these post-masks. Nonetheless, the dual-task deficit was small, and there was no stimulus processing tradeoff. Color-detection performance was consistent with parallel processing, even in the presence of strong backwards masks.

### Parallel and then serial processing

Our interpretation of the data is that the word recognition system has a parallel front end followed by a serial bottleneck (Reichle, Vanyukov, Laurent, & Warren, 2008; White et al., 2018, 2019). Two words can be encoded and visually processed in parallel, up to a point. Their sublexical (perhaps even orthographic) features can also be stored in a short-term memory trace. If masks do not arrive immediately after, then the lexical attributes of both words can be processed serially, one and then the other. But if the word-mask ISI is set to threshold, one word can be fully processed, but by then the mask has eliminated any stored information about the other word.

We propose that the serial bottleneck lies at or just before the stage of lexical access: when the visual form of the letter string is associated with a lexical entry in long-term memory. As evidence, we point to the fact that the serial model held even for lexicality judgments (Exp. 2), which measure the efficiency of lexical access without further semantic processing. Moreover, single-task accuracy rose with lexical frequency in Experiments 1 and 2, indicating that lexical access is facilitated for more familiar words. However, the dual-task deficit was actually larger for more frequent words, and the serial model held across the range of frequencies. We interpret that as meaning that two words cannot simultaneously reach the stage at which lexical frequency has an effect.

Our model of parallel visual processing and serial lexical access is consistent with recent neuroimaging evidence. We recorded fMRI activity while participants performed a semantic categorization task similar to Experiment 1, but with the words to the left and right of fixation. Our analysis focused on retinotopic visual cortex, where the responses evoked by each word are spatially separated, and subregions of the “visual word form area” (VWFA). The VWFA lies in ventral occipital-temporal cortex, is typically left-lateralized, and performs functions critical to reading (Dehaene & Cohen, 2011; Wandell, Rauschecker, & Yeatman, 2012).

We observed parallel processing of both words in retinotopic visual cortex, as well as in the posterior sub-region of the VWFA. This builds off of prior findings of unlimited-capacity parallel processing in visual cortex with a non-linguistic task (White, Runeson, Palmer, Ernst, & Boynton, 2017). However, in the anterior sub-region of the left hemisphere VWFA, neuronal responses were consistent with serial processing of single words after the bottleneck. Moreover, lexical frequency modulated BOLD response in this region for only attended words. We concluded that parallel processing of the two words extends through the visual system, up to a relatively late stage where the visual system and the language system intersect (White et al., 2019).

### Distinguishing parallel and serial models

Parallel and serial models of perceptual and cognitive processing are famously difficult to distinguish (Townsend, 1990). Key features of our approach are that we use backwards masks to limit processing time and measure accuracy and in order to test the viability of an “all-or-none” serial model. We now consider several possible challenges to that model.

First, could a fixed-capacity parallel model produce the stimulus processing tradeoff between the two sides (Fig. 3)? That is possible. Suppose that the observer does not consistently and evenly divide their parallel processing resources between the two sides. On some trials, they devote the majority of resources to the top word, and on the other trials, to the bottom word. That would produce a tradeoff between the two sides: on the trials when they get the top word correct, they are less likely to get the bottom word correct. However, to produce a tradeoff as large as we observed, the observer would have to distribute their resources so unevenly that they are effectively mimicking the all-or-none serial model, because one word is almost completely ignored.

Second, could other types of parallel models explain the AOCs we observed for semantic and lexical judgments? We generated predictions for two standard parallel models (unlimited-capacity and fixed-capacity) that account for many tasks. One could also imagine a parallel model that is more limited than the fixed-capacity model: dividing attention reduces the fidelity of the stimulus representations even below the level predicted by sharing a constant amount of information. Thus, the two stimuli are processed simultaneously, but due to some extra difficulty of dividing attention, the process is so poor that accuracy falls to the serial model’s prediction. Unlike the all-or-none serial model, this model requires ad hoc additions to fit the data. Moreover, it would not predict the stimulus processing tradeoffs that we observed, without even more ad hoc additions as described above. We look forward to future work that tests more complex parallel models that apply to tasks like ours by incorporating the temporal dynamics of recognition and decision.

Third, is it possible that the lexical and semantic attributes of two words are processed in parallel, but the serial bottleneck is for *conscious* identification of those attributes? Snell and Grainger (2019a) made that argument, in support of a “parallel cascaded” model of word recognition during reading (Wen, Snell, & Grainger, 2019). Our paradigm cannot definitively rule that out, although we remain agnostic as to whether the forced-choice responses in our experiments were based on conscious awareness or not. Indeed, some research supports the hypothesis that when a word is rapidly masked, semantic processing proceeds without conscious awareness (e.g., Holender, 1986). It is therefore possible that our participants were responding based on subliminal lexical information without a conscious percept of the stimulus identity, and even so, only had access to (unconscious) information about one of the two words. In other words, one hypothesis consistent with our data is that there is a serial bottleneck for lexical access even when there is no conscious awareness of the lexical information. The alternative hypothesis is that both words in our paradigm were processed to a lexical level prior to a filter at the stage of conscious awareness that is required to perform our explicit forced-choice task. The congruency effects we observed could be evidence of sub-threshold processing both words, such that the semantic category of one unconsciously influences the decision for the other. However, a serial model could also explain congruency effects by supposing biased guessing or selection errors.

Other authors have argued for parallel processing of multiple words on the basis of congruency effects observed when subjects judge a single fixated word that is flanked by other words that may be congruent or incongruent (Dallas & Merikle, 1976; Shaffer & LaBerge, 1979; Snell, Declerck, et al., 2018; Snell & Grainger, 2018; Snell et al., 2017; Underwood & Thwaites, 1982). Broadbent and Gathercole (1990) argued that such congruency effects do not necessarily imply automatic parallel processing of multiple words (see also Lachter et al., 2004). We also note that in the more recent experiments by Snell and colleagues showing congruency effects, the words were presented for 150–170 ms and not masked. The processing time available was therefore well above the thresholds we have measured here, and could have allowed for serial processing of all words within each trial, thus leading to congruency effects.

More experiments are required to distinguish between these various hypotheses about parallel versus serial semantic processing with and without conscious awareness. One approach to build upon the results reported here would be to measure subliminal semantic priming with pairs of words and directly assess conscious awareness of their identities.

Another form of interaction between simultaneously presented words is called a “migration error.” This phenomenon has been demonstrated by presenting subjects with two words, one on either side of fixation, that are then post-masked. The subject is then asked to report one or all of the letters within one of words. Interestingly, they sometimes report letters that were present in the other word. This type of “migration” error is sensitive to high-level lexical properties, and has been taken as evidence of parallel word recognition (McClelland & Mozer, 1986; Mozer, 1983; Snell & Grainger, 2019b). Again, however, the time between the onset of the words and the post-masks in those experiments was quite long, on the order of 200–500 ms. Given that in our experiments single words can be recognized with only 30–50 ms between word onset and mask onset (Table 1), it is possible that migration errors are due to confusion *after* both words were processed serially, within the time allowed in each display.

### Relation to natural reading and outstanding questions

Our experiments differ from natural reading in several important respects. First, both words were in the parafovea and not fixated directly. Second, the words were unrelated to each other and devoid of context. In natural reading, individual words are successively fixated, and attention shifts into the parafovea to begin processing the next words, which can be predicted to some degree based on the sentence context. In theory, sentence context could reduce the amount of information that readers must extract from each word for comprehension, and therefore allow for more parallel processing.

Indeed, the authors of one study argued that the effect of sentence context on the recognition of words in brief displays is evidence for parallel word processing (Snell & Grainger, 2017). In that study, sets of four words were displayed simultaneously for 200 ms and then masked. Again, our results suggest that this could be enough time to process multiple words serially, so the effect of sentence context in that experiment does not necessarily imply parallel processing. Beyond the differences in stimulus timing, the apparent discrepancy between studies may hinge on a better understanding of the nature of internal processing required for “recognition” in different contexts. The cognitive operations that underlie recognition as measured in our explicit forced choice tasks may differ from the operations required for efficiently comprehending sentences.

Future work can apply our tests to conditions more similar to natural reading. For instance, one of the two words could be placed directly at the center of gaze. The two words could also be related to each other (e.g., forming compound words or common phrases), or they could be embedded in sentence context. The results of such experiments would reveal how the reading circuitry operates within the confines of the severe capacity limits that we have documented here.

Other important questions for future research concern the specificity of the serial bottleneck. First, for written words, would other tasks that tap into sublexical orthographic or phonological features demonstrate parallel processing? Second, is the serial bottleneck demonstrated here specific to words, or common to all complex visual objects? For instance, is it possible to recognize two faces at once? Can two common objects in natural scenes be simultaneously identified? These questions are the focus of ongoing research.

## Conclusion

The experiments reported here demonstrate that for a range of conditions, the standard fixed-capacity parallel model overestimates how well subjects can simultaneously process the linguistic attributes of two written words. However, the same parallel model can account for judgments of text color. We propose that visual processing of two words begins in parallel, but a serial bottleneck lies at or just prior to lexical access.

### Electronic supplementary material

ESM 1 (PDF 374 kb)

## Appendix

### Models of capacity limits

In the attention operating characteristic plots, we compare dual-task accuracy to the quantitative predictions of three models of capacity limits. These are the same models as we used in a previous publication (White et al., 2018).

In all of the experiments reported in this article, each stimulus belongs to one of two categories. We designate one of those the “target” category: living things in Experiment 1, real words in Experiment 2, and colored letter strings in Experiment 3. The subject’s task is to report whether or not a particular stimulus is a target. We assume that the subject analyzes the stimulus on each side *i* by computing an estimate *E*_*i*_ of the evidence that the stimulus is a target. We assume that across trials of one condition, the *E*_*i*_ values for both sides are independent and identically distributed Gaussian variables. Within each trial, *E*_*1*_ and *E*_*2*_ are independent of each other.

Sensitivity *(d’*) depends on the mean difference in *E* between target-present and target-absent trials, relative to the across-trial variability in *E*. To make a judgment about each stimulus, E is compared against three criteria *c*_*1*_*, c*_*2*_*, c*_*3*_ to determine which of the four response keys to press.

We first label the measured single-task accuracy levels for the top and bottom stimuli *A*_*T1*_ and *A*_*B1*_, respectively (in units of area under the ROC curve). The three models then use these single-task accuracy levels to predict the dual-task levels *A*_*T2*_ and *A*_*B2*_.

1. *Unlimited-capacity parallel processing model*: This model assumes that the distribution of *E*_*i*_ is identical in the single-task and dual-task conditions. It therefore predicts no dual-task deficit:

$$ {A}_{T2}={A}_{T1}, $$

and

$$ {A}_{B2}={A}_{B1} $$

2. *Fixed-capacity parallel processing model*: This model assumes that the perceptual system extracts a constant amount of information from the display regardless of the condition (single-task vs. dual-task). In the dual-task condition, if that information is equally distributed between the two stimuli, only half as much information is available about each as in the single-task condition. One way to conceptualize fixed capacity is to assume that computing *E*_*i*_ (evidence in favor of the target category) depends on gathering sensory ‘samples’ from the stimulus (Shaw, 1980). All attended stimuli must share a fixed number *S* of samples that can be gathered from the whole display per unit time. The variability of *E*_*i*_ is inversely proportional to the number of samples assigned to stimulus *i*, which means that reducing the number of samples decreases sensitivity. As the proportion *q* of samples given to the bottom stimulus increases from 0 to 1, this model’s prediction traces out the black curve that connects the two single-task data points in the AOC plot. This curve is computed as follows. We first calculate *d’* for the top and bottom single-task conditions:
  $$ d{\prime}_{T1}=\sqrt{2}\ {F}^{-1}\left({A}_{T1}\right), $$

and

$$ d{\prime}_{B1}=\sqrt{2}\ {F}^{-1}\left({A}_{B1}\right) $$

where *F*^*-1*^ is the inverse of the normal cumulative distribution function.

We then assume that in the dual-task condition, the top stimulus receives *qS* samples, and the bottom stimulus receives the remaining (1-*q*)*S* samples, where 0<*q*<1. From signal detection theory, receiving a proportion *q* of samples changes *d’* for that stimulus by a factor √*q* (relative to the single-task condition when *q* = 1). Therefore, *d’* for each side in the dual-task condition is:

$$ d{\prime}_{T2}=\sqrt{q}\left(d{\prime}_{T1}\right), $$

and

$$ d{\prime}_{B2}=\sqrt{1-q}\left(d{\prime}_{B1}\right) $$

We then convert these d-prime measures into accuracy as proportions:

$$ {A}_{T2}=F\left(d{\prime}_{T2}/\sqrt{2}\operatorname{}\right), $$

and

$$ {A}_{B2}=F\left(d{\prime}_{B2}/\sqrt{2}\operatorname{}\right) $$

where *F* is the normal cumulative distribution function.

The parallel model can be generalized to predict less severe deficits, by assuming that the total number of samples available in the dual-task condition is more than the number (*S*) in the single-task condition. That is, the total number of samples shared between the two locations is *aS*, where 1=<*a*<=2. Increasing *a* pushes the predicted curve into the upper right corner, eventually meeting the prediction of the unlimited capacity model when *a* = 2.

3. *All-or-none serial-processing model*: This model assumes the same stimulus representations and decision rule as the parallel models. Like in the unlimited-capacity parallel model, the distributions of *E* are identical in single- and dual-task conditions. The difference is that this model assumes that the subject processes only one stimulus. They do not have time to even start processing the second, and therefore must guess when asked about it. As the proportion of trials *v* in which the top stimulus is processed increases from 0 to 1, this model’s prediction traces out the diagonal black line in the AOC plot.

To generate that prediction, we first present the model in a simplified form, with accuracy levels computed as mixtures of probabilities (in units of proportion area under the ROC curve or proportion correct). Dual-task accuracy for the top stimulus is:

$$ {A}_{T2}={A}_{T1}\ v+ 0.5\left( 1-v\right). $$

The second term in that equation reflects the fact that the participant must guess (with probability correct 0.5) on the (1-*v*) proportion of trials in which the top stimulus is not processed at all. The top and bottom sides trade off linearly, so dual-task accuracy for the bottom stimulus is:

$$ {A}_{B2}={A}_{B1}\left( 1-v\right)+ 0. 5v. $$

The serial model so far has been presented in a simplified form so that it can generate the AOC directly by computing mixtures of probabilities. The units can either be considered proportion under the ROC curve (A_g_) or proportion correct. Key to this simplified form is that accuracy for the side that is *not* processed on dual-task trials is set to 0.5. That assumes that the observer makes a random guess, and an unbiased estimate of the probability correct is 0.5.

We can make the model more complex and realistic by simulating trials using signal detection theory, drawing values of evidence *E*_*i*_ for each side *i*. Whenever side *i* is attended and processed, the value *E*_*i*_ is drawn from either the target-absent or target-present distribution. Those distributions are separated by *d’*, the discriminability for that stimulus in the single-task condition. Whenever side *i* is *not* processed, *E*_*i*_ is drawn from a “default” distribution midway between the target-present and target-absent distributions (with the same standard deviation). Then the observer makes a decision by comparing *E*_*i*_ to the same criteria as in the other conditions, but in this case *E*_*i*_ conveys no information about whether the stimulus was a target or not.

To generate the dual-task stimulus processing tradeoff predicted by the all-or-none serial model (Fig. 3), we simulate thousands of trials. The model takes as input the single-task *d’*, and *v* (the proportion of dual-task trials when the top stimulus is processed). For each trial, it draws *E*_*1*_ and *E*_*2*_ from appropriate Gaussian distributions, with means that depend on the category (target or non-target) of each stimulus and whether the top one was processed (on *v* proportion of trials) or whether the bottom one was processed (on 1-*v* proportion of trials). It then generates the subject’s response for each stimulus by comparing *E*_*1*_ and *E*_*2*_ to a set of criteria that span the range of *E*. Finally, we analyze those data in the same way as we analyze our real data: sorting the dual-task responses according to whether the other response on the same trial was correct or incorrect, and computing area under the ROC curve for each.

The predicted curve on Fig. 3 was computed by varying *d’*. The difference between trials when the other side’s response was correct vs. incorrect increases as *d’* rises. This prediction was found to be invariant to changes in *v.* Note that we also ran a similar simulation for the fixed-capacity parallel model, which always predicts no effect of the other side’s accuracy (i.e., its prediction lies along the identity line in Fig. 4).

Finally, the serial model can be generalized to account for less severe dual-task deficits by assuming that on some fraction *w* of dual-task trials, *both* sides are fully processed (with the same sensitivity as in the single-task conditions). Such a model is no longer a pure “all-or-none” serial model. The resulting dual-task accuracy is a mixture of trials in which only one stimulus is processed and no information is acquired about the other, and in which both stimuli are fully processed.

$$ {\displaystyle \begin{array}{l}{A}_{T2}=w{A}_{T1}+\left(1-w\right)\left({A}_{T1}v+0.5\left(1-v\right)\right)\\ {}{A}_{B2}=w{A}_{B1}+\left(1-w\right)\left({A}_{B1}\left(1-v\right)+0.5v\right)\end{array}} $$

In this generalized model, *v* can be interpreted as the proportion of trials in which the top stimulus is processed “first.” On only *w* fraction of trials is the “second” stimulus processed at all.
