# Supplementary material for: Visual word recognition: Evidence for a serial bottleneck in lexical access
Source: Atten Percept Psychophys. 2019 Dec 12;82(4):2000–17. doi: 10.3758/s13414-019-01916-z (PMC7297702; doi:10.3758/s13414-019-01916-z)
Supplement: Supplementary file 1 — (PDF 374 kb) [file 13414_2019_1916_MOESM1_ESM.pdf]

Supplementary Material for White, Palmer & Boynton (2019):
Visual word recognition: evidence for a serial bottleneck in lexical access

Individual subject Attention Operating Characteristics

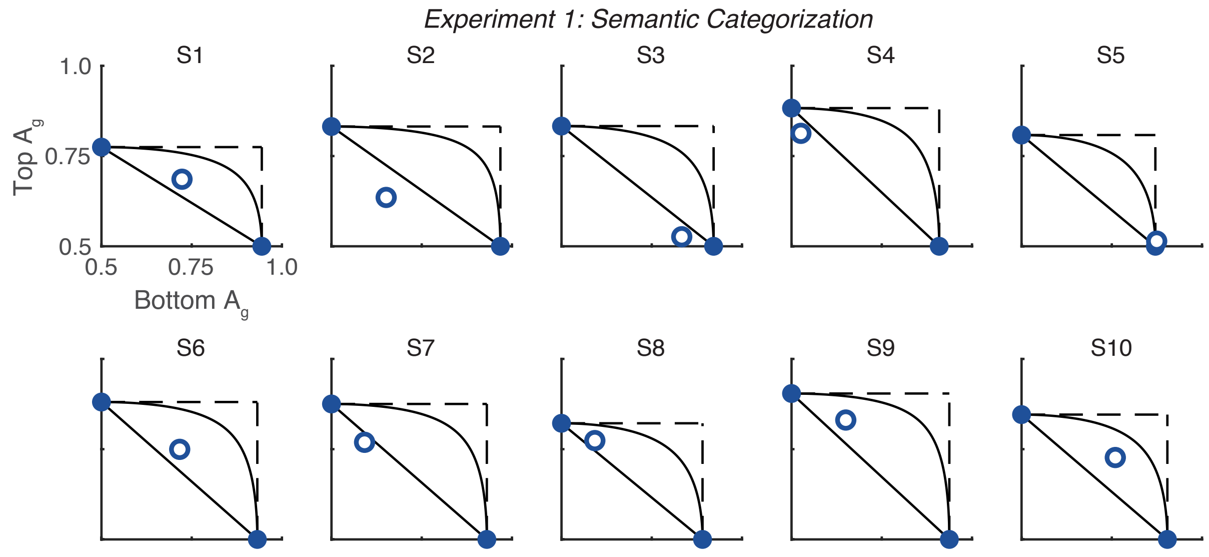

Figure S1: Attention Operating Characteristics for individual subjects in Experiment 1 (semantic categorization), collapsing over both mask types. Format as in Figure 2; closed symbols are single-task accuracy levels and open symbols are dual-task accuracy levels.

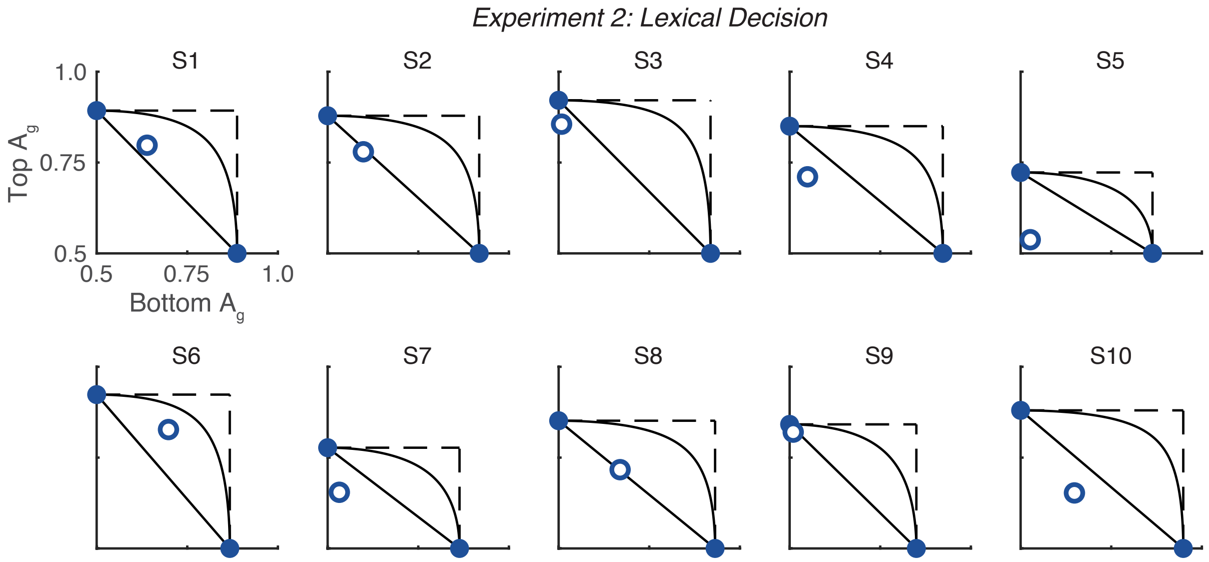

Figure S2: Attention Operating Characteristics for individual subjects in Experiment 2 (lexical decision). Format as in Figure S1.

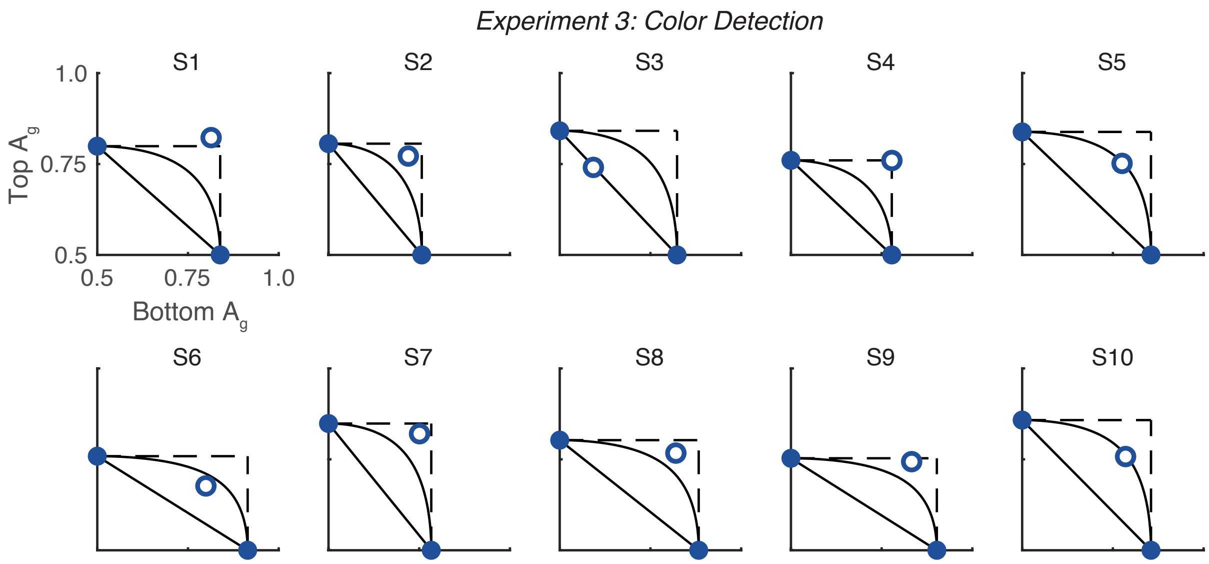

Figure S3: Attention Operating Characteristics for individual subjects in Experiment 3 (color detection). Format as in Figure S1.

Dual-task accuracy correlations

In the main text, we demonstrate the stimulus processing tradeoff by analyzing accuracy conditional on the accuracy of the response to the other side on dual-task trials. Here we present a related analysis: the correlation between the accuracies of responses to the two sides (Bonnel & Prinzmetal, 1998; Ernst, Palmer, & Boynton, 2012; Lee, Koch, & Braun, 1999; Sperling & Melchner, 1978). The stimulus processing tradeoffs we observed in Experiments 1 and 2 (higher accuracy when the other side's response was incorrect) would predict a negative accuracy correlation.

In Experiment 1, the across-subject mean correlation coefficient r was -0.043 ± 0.010 ($t(9) = 4.172$, $p=0.002$, CI = [-0.064 -0.025]). In Experiment 2, mean $r = -0.025 \pm 0.016$, $t(9) = 1.54$, $p=0.158$, CI = [-0.053 0.007]. In Experiment 3, mean $r = 0.027 \pm 0.020$, $t(9) = 1.37$, $p=0.205$, CI = [-0.010 0.063].

Altogether, the correlations are consistent with the stimulus processing tradeoff patterns reported in the main text, although noisier. As we have argued previously (White, Palmer, & Boynton, 2018), the correlations could be contaminated by effects of the other side's response on decision criterion. In contrast, the conditional analysis of stimulus trade-offs (Figure 3) computes area under the ROC curve as a bias-free measure of accuracy.

Length and frequency effects on accuracy

We first analyzed accuracy in each experiment as a function of the number of letters in the string being judged, as shown in the top row of **Figure S4.** For these analyses, we use the sensitivity measure *d′* rather than A_g, because we need to evaluate the interactions between word length and cue condition (single-task vs. dual-task). A_g is a proportion and therefore not ideal for analyzing interactions when there are also main effects (Loftus, 1978). In contrast, *d′* can be assumed to scale linearly with the signal-to-noise ratio of the stimulus representations.

In Experiment 1, single-task *d′* increased from 4-letter to 6-letter words by an average of 0.47 ± 0.13 (t(9)=3.64, p=0.005, CI = [0.26 0.74]). The opposite happened in Experiment 2: single-task *d′* decreased from 3-letter to 5-letter strings, by an average of 0.65 ± 0.09 (t(9)=7.07, p=0.0001, CI = [0.47 0.81]).

In general, skilled readers show little effect of word length on recognition performance (Nazir, 2007), but decrements for longer words have been reported when the stimuli are not fixated directly (Bub & Lewine, 1988; Ellis, 2004). The negative effect of increasing length on accuracy in the lexical decision task (Expt. 2) is therefore not entirely surprising. The *positive* effect in the semantic categorization task (Expt. 1) is more difficult to explain. Perhaps the subjective discriminability of the 'living' and 'nonliving' categories increases as word length increases.

Dual-task *d′* (open symbols in **Figure S4**) was affected by length in a similar manner as single-task *d′* in each experiment. But is the relative dual-task deficit smaller for shorter words? That would predict a particular two-way interaction between cue condition (single-task vs. dual-task) and length. To the contrary: In Experiment 1, there was no significant interaction (F(2,18)=0.47, p=0.63), although there were significant main effects of length (F(2,18)=14.3, p=0.0002) and cue (F(1,9)=573, $p<10^{-7}$). The dual-task deficit on *d′* was equivalent for short and long words (mean difference in deficits = 0.07 ± 0.09, t(9)=0.71, p=0.49, [-0.11, 0.23]).

In Experiment 2, there was a significant interaction between length and cue condition (F(2,18)=13.0, p=0.0003), in addition to main effects of length (F(2,18)=32.0, $p<10^{-4}$)and cue (F(1,9)=165.4, $p<10^{-7}$). However, the mean dual-task deficit was 0.46 ± 0.09 *d′* units *larger* for the shortest than for the longest words (t(9)=5.47, p=0.0004, CI = [0.33 0.66]. That runs counter to the prediction that short words are easier to process in parallel.

In Experiment 3 (color detection), single-task *d′* increased from 3-letter to 5-letter strings, by an average of 0.40 ± 0.12 (t(9)=3.30, p=0.009, CI = [0.22 0.69]). The longer the targets, the more colored letters were present, increasing the probability of detection. There was no significant interaction between length and cue condition (F<1). The dual-

task deficit was slightly but not significantly smaller for the shortest than and longest words (mean difference = 0.15 ± 0.14, t(9)=1.08, p=0.31, CI = [-0.03 0.57]).

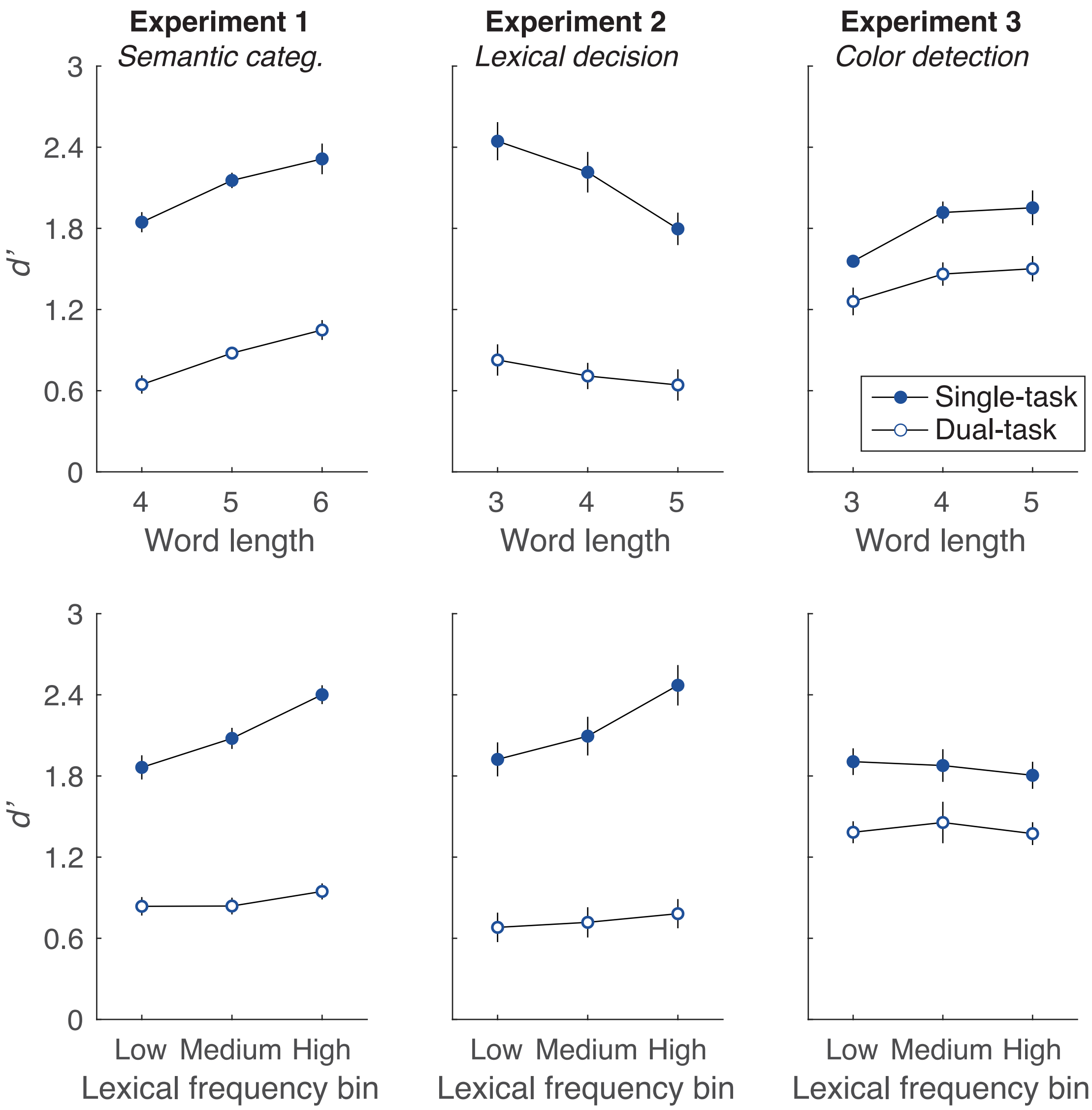

Figure S4: Accuracy expressed as *d'* plotted a function of string length (**top row**) and lexical frequency bin (**bottom row**), for the single-task and dual-task conditions separately, in each experiment. Error bars show ±1 SEM (N=10).

We next analyzed *d'* as a function of lexical frequency (measured as occurrences/million), by sorting each stimulus set into three equally sized bins. In Experiment 1, the frequency bins were: low (0.06 – 3.4 per million); medium (3.4 – 14.5), and high (14.5 – 539). For the stimulus set used in Experiments 2 and 3, the bins were:

low (3.4 – 12.2); medium (12.2 – 50.1); and high (50.1 – 872). Note that in Experiment 2 (lexical decision) it was not possible to directly compute *d′* in each frequency bin. All pseudowords have 0 lexical frequency, so we cannot analyze the rate of false alarms (incorrectly reporting that a pseudoword was a real word) in each bin. We therefore first compute the false alarm rates from pseudoword trials and assume they are constant across frequency bins**.** See below for an analysis of hit rates separately, which show a very similar pattern.

As shown in the bottom row of **Figure S4,** single-task *d′* in Experiments 1 and 2 increased with lexical frequency (both $F(2,18)>14$, $p<0.001$). More common words are easier to recognize, even with focused attention. The mean difference in *d′* between the high and low bins was 0.54 ± 0.12 in Experiment 1 ($t(9)=4.46$, $p=0.0016$, CI = [0.31 0.75]), and 0.55 ± 0.09 in Experiment 2 ($t(9)=5.82$, $p=0.0003$, CI = [0.36 0.71]).

The key question is whether the dual-task deficit is smaller for high-frequency words. In fact, the dual-task deficit was *larger* for words in the high-frequency bin than in the low frequency bin, by an average of 0.43 ± 0.13 in Experiment 1 ($t(9)=3.12$, $p=0.011$, CI = [0.19 0.68]) and 0.45 ± 0.09 in Experiment 2 ($t(9)=5.82$, $p=0.0003$, CI = [0.31, 0.59]). Those differences were reflected in significant interactions between cue condition and frequency bin (Expt. 1: $F(2,18)=5.33$, $p=0.015$; Expt. 2: $F(2,18)=17.18$, $p=0.00007$). Therefore, it does not seem that pairs of common words can be processed in parallel.

In Experiment 3 (color detection), single-task d′ was not significantly affected by frequency bin ($F<1$), and there was no interaction with cue condition ($F<1$). The dual-task deficit did not reliably differ between the low- and high-frequency bins (mean difference = 0.09, ± 0.12; $t(9)=0.76$, $p=0.47$, CI = [-0.11 0.34]).

Hit rates as a function of lexical frequency in Experiment 2

In the above analysis of lexical frequency effects on *d′* in Experiment 2, we had to assume that the false alarm rates (incorrectly reporting pseudowords to be real words) were constant across frequency bin. As a complementary analysis that doesn't rely on that assumption, we analyzed hit rates (correctly reporting real words) as a function of the lexical frequency bin, in the single-task and dual-task conditions separately.

The question is whether the dual-task deficit on hit rates becomes less severe as lexical frequency increases. As shown in **Figure S5**, the opposite occurred. Hit rates rose as a function of frequency in the single-task condition faster than in the dual-task deficit. As a result, the average dual task-deficit *increased* significantly from the low frequency bin (0.25 ± 0.03) to the high bin (0.31 ± 0.02; comparison across bins: $t(9)=2.86$, $p=0.019$, CI = [0.03, 0.11]). Therefore, lexical decision for two words in parallel does not become easier as their lexical frequency increases.

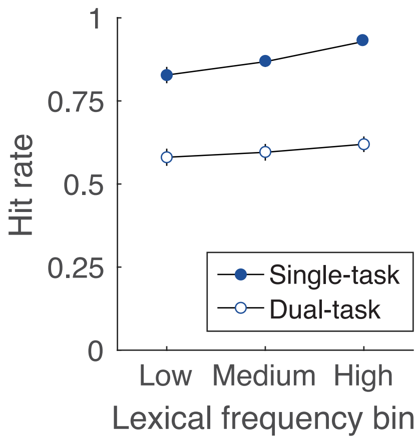

Figure S5: Hit rates in Experiment 2 (lexical decision task) as a function of the words' lexical frequency bin. Error bars show ±1 SEM (N=10). The hit rate is the proportion of correct responses to real words.

References

Bonnel, A.-M., & Prinzmetal, W. (1998). Dividing attention between the color and the shape of objects. *Perception & Psychophysics*, *60*, 113–124.

Bub, D. N., & Lewine, J. (1988). Different Modes of Word Recognition Visual Fields. *Brain and Language*, *188*, 161–188.

Ellis, A. W. (2004). Length, formats, neighbours, hemispheres, and the processing of words presented laterally or at fixation. *Brain and Language*, *88*, 355–366.

Ernst, Z. R., Palmer, J., & Boynton, G. M. (2012). Dividing attention between two transparent motion surfaces results in a failure of selective attention. *Journal of Vision*, *12*, 1–17.

Lee, D. K., Koch, C., & Braun, J. (1999). Attentional capacity is undifferentiated: Concurrent discrimination of form, color, and motion. *Perception & Psychophysics*, *61*, 1241–1255.

Loftus, G. R. (1978). On interpretation of interactions. *Memory & Cognition*, *6*, 312–319.

Nazir, T. A. (2007). Traces of Print Along the Visual Pathway. *Reading as a Perceptual Process*, 3–22.

Sperling, G., & Melchner, M. J. (1978). The attention operating characteristic: examples from visual search. *Science*, *202*, 315–318.

White, A. L., Palmer, J., & Boynton, G. M. (2018). Evidence of serial processing in visual word recognition. *Psychological Science*, *29*, 1062 –1071.
